# Ultralow temperature cryoablation using near‐critical nitrogen for cavotricuspid isthmus‐ablation, first‐in‐human results

**DOI:** 10.1111/jce.15142

**Published:** 2021-07-09

**Authors:** Martijn N. Klaver, Tom J. R. De Potter, Konstantinos Iliodromitis, Alexander Babkin, David Cabrita, Davide Fabbricatore, Lucas V. A. Boersma

**Affiliations:** ^1^ Department of Cardiology St. Antonius Hospital Nieuwegein The Netherlands; ^2^ Department of Cardiology Onze‐Lieve‐Vrouw Ziekenhuis Aalst Belgium; ^3^ Adagio Medical Laguna Hills California USA; ^4^ Department of Cardiology Amsterdam University Medical Centers The Netherlands

**Keywords:** cavotricuspid isthmus, cryoablation, first‐in‐human, near‐critical nitrogen, ultralow temperature

## Abstract

**Introduction:**

Cryoablation has evolved as a safe alternative to radiofrequency ablation in the treatment of several supraventricular arrhythmias and has potential advantages, yet is limited by the properties of the cryogen used. We investigated a novel ultralow temperature cryoablation (ULTC) system using nitrogen near its liquid‐vapor critical point as a freezing source, achieving temperatures as low as ‐196 degrees Celsius in a long linear catheter with a continuous energy release. Initial safety, procedural and efficacy outcomes of ULTC are described in patients undergoing cavotricuspid isthmus (CTI) ablation.

**Methods and Results:**

The Cryocure studies (NCT02355106, NCT02839304) are prospective, single‐arm, multi‐center, first‐in‐human clinical studies in 17 patients with atrial flutter (AFL) and 13 patients with atrial fibrillation (AF). A total of 30 patients, mean age 65 ± 8 years old and 67% male, were enrolled and underwent ablation of the CTI. Acute success, defined as the confirmation of stable bidirectional conduction block across the CTI, was achieved in all 30 patients. After 12 months of follow‐up, 14 out of 17 AFL patients remained free from any AFL. One (3.3%) procedure‐related but not device‐related serious adverse event was reported, involving transient inferolateral ST‐elevation associated with temporary AV conduction block.

**Conclusion:**

In this first‐in‐human clinical study the safety and performance results demonstrate the capabilities of ultralow temperature near‐critical nitrogen as an effective energy source for CTI ablation. Ongoing, larger, studies should confirm our findings and evaluate the capabilities to create linear and focal transmural lesions in other arrhythmias.

**Abbreviations**
AF: atrial fibrillation
AFL: atrial flutter
AV: atrioventricular
BCB: bidirectional conduction block
CC1: Cryocure 1
CC2: Cryocure 2
CN_2_
: nitrogen (critical pressure)
CTI: cavotricuspid isthmus
DSMB: data safety monitoring board
EPS: electrophysiology studies
GA: general anesthesia
IQR: interquartile range
N_2_O: nitrous oxide gas
NOAC: non‐vitamin K oral anticoagulants
RF: radiofrequency
ULTC: Ultralow temperature cryoablation
VKA: vitamin K‐antagonists

## 1 INTRODUCTION

Ablation treatment has acquired a prominent role in the treatment of supraventricular arrhythmias, leading to an increasing demand for operating rooms and operator time. New technologies aim to improve the efficacy, speed and durability of ablation treatment. Cryoablation has evolved as a safe alternative to radiofrequency (RF) ablation in the ablation of supraventricular arrhythmias.^1^, ^2^, ^3^, ^4^, ^5^, ^6^ Potential advantages of cryothermal energy are catheter stability due to adherence to myocardial tissue, the ability of reversible cryomapping mitigating the risk of permanent damage to critical structures like the atrioventricular (AV) node, a low risk of thrombus formation and systemic embolization, and a low probability of myocardial perforation due to the preservation of tissue architecture.^1^, ^2^, ^5^, ^7^, ^8^, ^9^, ^10^ Yet, efficacy of cryoablation using gaseous cryogens may be hampered by physiological reversibility of cellular injury when ablation times are too short or freezing temperatures not low enough, allowing thicker areas of myocardium to recover from freezing injury and recurrence of arrhythmias to occur.^11^, ^12^

The efficiency and speed of cryoablation is first and foremost determined by cryogen that is used in the ablation catheter. Classic cryoablation systems have used gaseous cryogens (predominantly nitrous oxide gas [N_2_O]), which have a much lower thermal capacity due to their small density when compared to liquid cryogens. This limits the catheters' overall cooling power, cooling speed, minimum temperature and effective cooling surface, while resulting in a massive gas consumption, mandating multiple applications (freeze‐thaw‐cycles) of several minutes.^12^, ^13^, ^14^, ^15^ Although liquid cryogens possess much higher thermal conduction and greater heat capacity than N_2_O, they are difficult to handle in endovascular catheters and considered unreliable and unsafe. Nitrogen near its liquid‐vapor critical point (near‐critical nitrogen) provides a unique solution that can be used in cardiac applications. Owing to its very low viscosity, it can be flowed down tiny channels while providing a freezing capacity that can overcome endovascular heat sinks. This new concept of near‐critical cooling makes it possible to maintain an uninterrupted flow of a cryogen close to its critical state along the full catheter length. It also solves issues related to slow “start‐stop” times present in liquid cryogen flow systems, limited to larger probe sizes.^12^

Recently, Adagio Medical has developed a novel cryoablation system using near‐critical nitrogen as a freezing source, achieving temperatures as low as −196°C in a 9F linear catheter with continuous energy release. This could overcome many of the efficiency and speed limitations with prior cryoablation platforms. Preclinical data demonstrated that ultralow temperature cryoablation (ULTC) is highly effective and capable of creating durable, transmural and contiguous atrial and ventricular lesions in an in vivo model in large animals.^16^ In this first‐in‐human study, we describe the initial safety and efficacy outcomes using the Adagio ULTC system to create durable bidirectional cavotricuspid isthmus (CTI) conduction block.

## 2 METHODS

### 2.1 Study population

The Cryocure 1 (CC1, NCT02355106) and Cryocure 2 (CC2, NCT02839304) clinical studies are prospective, single‐arm, multi‐center, first‐in‐human clinical studies to assess the initial safety and performance of the Adagio Medical Cryoablation System. The treatment population consisted of patients from CC1 and CC2 in which ablation of the CTI was performed. CC1 enrolled patients with a typical atrial flutter (AFL) pattern on ECG to undergo CTI alone. In CC2, patients with atrial fibrillation were enrolled and ablation of the CTI was performed as an adjunct target on top of left atrial ablation targets to obtain freedom of atrial fibrillation (AF), neither of which are the subject of this manuscript. Between January 2015 and May 2015 and between April 2017 and February 2019, all patients were enrolled in the two participating hospitals, “St. Antonius Ziekenhuis,” Nieuwegein, The Netherlands and the “Onze‐Lieve‐Vrouw Ziekenhuis,” Aalst, Belgium. The study protocol was approved by the Ethics committee, regulatory authorities and the institutional review board of both hospitals. All patients gave written informed consent. The medical history was reviewed, together with a physical exam to confirm inclusion and exclusion criteria.

### 2.2 The Adagio atrial flutter cryoablation system

The Adagio Cryoablation System consists of the Cryoablation Console and the Cryoablation Catheter. Treatment is achieved by ablating (or isolating) the arrhythmogenic tissue in contact with a distal freezing element of the catheter. During the treatment, the freezing element is cooled to cryogenic temperatures resulting in necrosis (ablation) of the tissue in contact with the catheter. The Adagio Catheter may create both linear (up to 54 mm) and focal lesions, depending on the length of the freezing element extended outside of the sheath.

The Adagio Cryoablation Console (Console) is a stand‐alone, electronic device providing the cryoablation energy and control mechanisms for the System. The delivery of thermal energy is created by pressurizing nitrogen to critical pressures (CN_2_), cooling it down to approximately ‐196 degrees Celsius, using a liquid nitrogen heat exchanger and delivering the energy to a freezing element at the distal end of the catheter. This process is created and controlled within the Console.

**Figure 1:**
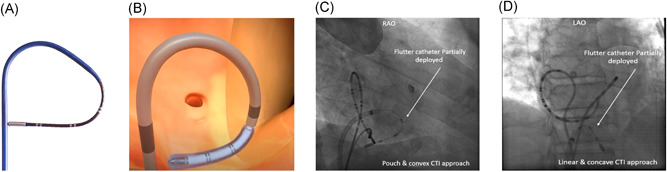
Adagio cryoablation catheter and fluoroscopic images of catheter positioning. (A) Photograph and (B) illustration of the Adagio medical flutter catheter using a convex approach. (C) Fluoroscopic visualization in a right anterior oblique projection of the convex approach and (D) the linear approach is visualized on fluoroscopy in a left anterior oblique projection

The Adagio Cryoablation catheter is a single‐use, sterile device that connects directly to the console and provides the delivery mechanism of the cryoenergy to the targeted tissue. The circumference of the catheter is 9.0 French. A thermocouple at the distal portion (freezing element) provides temperature readouts for operational status. In addition, the cryoablation catheters include electrodes along the freezing zone to enable verification of tissue contact and fluoroscopic landmarks for correct positioning of the catheter's freezing element within the right atrium (Figure 1). No mapping system is required. The catheter includes a closed, internal lumen where the non‐insulated portions (37–54 mm freezing element) can achieve cryoablation temperatures. An active vacuum channel surrounds the closed, internal lumen insulating the nonfreezing portions from cold temperatures, preventing all surfaces, other than the freezing zone, from developing cold temperatures. The catheter connects to the Adagio Cryoablation Console through a multi‐channel locking connector at the proximal end. To gain access to the inner walls of the beating heart, a commercially available guiding sheath was placed within the vasculature via a percutaneous femoral vein puncture as per normal vascular access procedures (Seldinger technique). The guiding sheath provides the conduit for the Cryoablation catheter to be placed near the targeted tissue to be ablated. The size and geometry of the catheter freezing zone (distal tip) can be customized to suit the desired target for ablation using a stylet. The catheter can be used in a linear convex, concave or circular shape, as well as focal. For CTI ablation, using a linear convex or concave approach, it is designed to easily contact and cover the CTI tissue, allowing for a single freeze once appropriate contact is made between the distal tip of the catheter and the CTI. The convex approach may overcome pouches along the CTI. The correct catheter position within the CTI is verified by using both fluoroscopy and the catheter electrodes, which enable verification of tissue contact and correct location of the cryoablation element within the right atrium. This makes its positioning easier, shortens the procedure time, and eliminates the need for an additional diagnostic catheter. The use of an electro anatomical mapping system is possible but not mandatory.

### 2.3 Electrophysiology study and ablation

Subjects were treated under conscious sedation or general anesthesia (GA) and prepared for ablation according to local standard. The procedures were performed under continued oral anticoagulation with vitamin K‐antagonists (target international normalized ratio between 2.0 and 3.0) or uninterrupted non‐vitamin K oral anticoagulants. All patients were then given an intravenous heparin bolus based on their weight. Once the cryoablation catheter was placed at the target ablation sites, freezes of one minute (limited to two minutes) in duration were performed along the CTI. Applications were continued until there was clear evidence of bidirectional conduction block (BCB). Repeat documentation of BCB was performed at a minimum of 30 min following the last ablation. BCB was confirmed using standard pacing methods and considered stable BCB if no conduction recovered. If conduction recovered, additional ablations were allowed, and the process for BCB verification at 30 min postablation was reinitiated. The use of isoproterenol for additional assessment of isthmus conduction was allowed at the investigators' discretion. At the completion of all ablation and mapping procedures, catheters and sheaths were removed, and hemostasis was obtained following the institutions standard of care. There were no pre‐specified requirements for length of stay or resumption of medications including, anticoagulation or antiarrhythmic drug therapy.

### 2.4 Post‐ablation management and follow‐up

All patients were monitored in hospital for 24 h. In the follow‐up phase of the study, two clinic visits were scheduled, followed by standard of care follow‐up until 12 months after the ablation procedure. During the clinic visits, a medical history, physical examination and 12‐lead ECGs were examined for an evaluation of patient arrhythmia status and any evidence of adverse events. Holter monitoring or event monitoring was performed when symptoms occurred and was left at the discretion of the treating physician. Additional follow‐up was collected up to 12 months after the procedure according to the hospitals' standard of care. All ECGs, continuous ECG monitoring and repeat electrophysiology studies (EPS) were reviewed for recurrent arrhythmias.

### 2.5 Outcome measures

The primary objective of the study was to provide data on the safety of ULTC using the Adagio medical device for CTI ablation. All safety events were classified by the severity of the event, the relationship of the event to the study device or procedure and whether the event or severity of the event was anticipated or unanticipated. All serious adverse events, unanticipated events and events of an unexpected severity were investigated by the Independent Data Safety Monitoring Board (DSMB) to determine the relationship to the investigational device. Serious adverse events were classified according to the MEDDEV definitions.

The secondary objective of the study was to access the performance of ULTC using the Adagio catheters. Acute success was defined as the confirmation of complete bidirectional conduction block across the cavotricuspid isthmus at the end of the cryoablation procedure after a minimum of 30 min following the last CTI freeze (stable BCB). The procedural characteristics, i.e. total procedure duration, fluoroscopy time and catheter dwell time were only collected for the CC1 patients (AFL). Ablation characteristics, i.e. cumulative freeze duration, number of freezes and length of each freeze, time until block and freezes until block were collected in all CC1 and CC2 patients (AFL and AF). When repeat EPS was performed during follow‐up, CTI conduction was evaluated and findings obtained (AFL and AF). Long‐term success was defined as the number of patients free from recurrent AFL on ECG at 12 months. Long‐term success was only collected in CC1 patients (symptomatic AFL).

### 2.6 Data collection

Data collected at all visits were entered through a web‐based electronic data capture system. The data were monitored following ISO 14155, Good Clinical Practice and in compliance with the Clinical Monitoring Plan. Maintenance of the study database was performed by the sponsor. As needed, data clarification requests were issued to sites and resolved to completion. The data underlying this article were provided by the sponsor. Data will be shared on request to the corresponding author with permission of the sponsor.

### 2.7 Statistical analysis

Descriptive statistics for all variables were applied. For continuous variables, mean and standard deviation or median and interquartile range, minimum and maximum were reported. For ordinal and categorical variables, counts and percentages were applied.

## 3 RESULTS

In a total of 30 patients, CTI ablation was performed; 17 from the CC1 cohort and 13 from the CC2 cohort (mean age was 65 ± 8 years old and 67% male). All patient characteristics are shown in Table 1. Procedural data are summarized in Table 2. CC1 patients were the first to be treated with the cryoablation system, while the CC2 patients were all part of a subsequent study.

**Table 1:** Baseline characteristics

**Characteristics**	**Result**
CTI only procedure	17 (56.7%)
CTI adjunct procedure	13 (43.3%)
Mean age	64.5 ± 7.6 years
Male gender	20 (66.7%)
Mean left ventricular ejection fraction	55% ± 6%
**Medical history**	
Hypertension	16 (53.3%)
Diabetes	4 (13.3%)
Coronary artery disease	5 (16.7%)
Prior stroke/TIA	1 (3.3%)
Congestive heart failure	1 (3.3%)
Significant valvular disease	1 (3.3%)
Cardiomyopathy	4 (13.3%)
Ischemic	1 (3.3%)
Hypertrophic	3 (10.0%)

Abbreviations: CTI, cavotricuspid isthmus; TIA, transient ischemic attack.

**Table 2:** Procedural characteristics

**Procedure data CC1 (*n* = 17)** a	**Result**
Procedure time (incl. 30‐minute waiting time) (minutes)^a^	85 ± 16 (54–116)
Fluoroscopy time (minutes)^a^	12 ± 5 (4–20)
Catheter dwell time (minutes)^a^	54 ± 14 (37–84)
**CTI ablation data (*n*** = **30)**	
Total CTI freeze time (minutes), median (IQR)	4.0 (2.9–5.3) (2.0–12.0)
Number of freezes per patient (incl. bonus freeze), median (IQR)	4 (2–5) (2–12)
Average duration per freeze (minutes), median (IQR)	1.1 (1.0–2.0) (0.55–2.0)
Freeze time until BCB (minutes), median (IQR)	1.7 (0.67–4.0) (0.23–8.7)
Number of freezes until BCB, median (IQR)	3 (1–4) (1–11)
Number of subjects with BCB during first freeze	12 (40%)

Abbreviations: AFL, atrial flutter; BCB, bidirectional conduction block; CTI, cavotricuspid isthmus; IQR, inter quartile range.

^a^ Data was only available for CryoCure 1 patients.

### 3.1 Safety results

Safety data was available in all patients. There was one serious adverse event reported among 30 patients (Table 3). The event consisted of transient ST‐elevation in the inferolateral limb leads of the 12‐lead ECG associated with a temporary AV conduction block, during a procedure in which GA was used. No medications were administered, and the ECG changes resolved spontaneously within a minute, including normal AV conduction. At the completion of the procedure, the patient was awake and free of symptoms. The patient was discharged the following morning as per standard hospital protocol postablation. The discharge and follow‐up ECGs showed sinus rhythm with normal AV‐conduction. The DSMB classified the event as serious, severe in intensity, and related to the procedure but not related to the device. The cause of the event was attributed to vasospasm of the right coronary artery due to a lateral placement of the cryoablation catheter.

**Table 3:** Adverse events

**Event term**	**SAE**	**Relationship to procedure**	**Relationship to device**	**Severity**
Coronary artery spasm	Yes	Yes	No	Severe

Abbreviation: SAE, severe adverse event.

### 3.2 Performance results

Acute success, defined as the confirmation of complete bidirectional conduction block across the CTI at the end of the cryoablation procedure after a minimum of 30 min waiting time following the last freeze, was achieved in 30 out of 30 patients (100%). No patients showed conduction recovery after the 30‐minute waiting time. During follow‐up of the CC1 group, three patients presented with recurrence of documented typical AFL after 3, 9 and 12 months respectively (Table 4). Repeat EPS was performed in 2 out of 3 patients, which confirmed recurrence of CTI‐conduction and inducible CTI‐dependent flutter for which repeat‐ablation was performed in both patients. The third patient did not undergo repeat EPS, and recovery of CTI conduction is unknown. Overall, after 12 months of follow‐up, 14 out of 17 patients remained free from typical atrial flutter (82.4%). In 10 of 30 patients repeated EPS was performed (8 in the CC1 group, 2 in the CC2 group) for recurrent AF or AFL, allowing reassessment of conduction over the CTI. In 7 out of 10 patients (70.0%) EPS demonstrated durable bidirectional block after a median of 11 [6, 7, 8, 9, 10, 11, 12] months. Recurrent conduction was seen in two symptomatic patients with documented return of AFL, both from the CC1 group and in one patient from the CC2 group, where no AFL was documented.

**Table 4:** Long‐term performance

	**Discharge** **AFL free**	**3 Months** **AFL free**	**6 Months** **AFL free**	**9 Months** **AFL free**	**12 Months** **AFL free**	**Repeat EPS**	**Durable CTI block**
CC1 patients (17)	17/17, 100%	16/17, 94.1%	16/17, 94.1%	15/17, 88.2%	14/17, 82.4%	8/17, 47.1%	6/8, 75%
CC2 patients (13)	13/13, 100%					2/13, 15.4%	1/2, 50%

Abbreviations: AFL, atrial flutter; CTI, cavotricuspid isthmus; EPS, electrophysiology study.

## 4 DISCUSSION

This is the first‐in‐human clinical trial evaluating the use of near critical nitrogen as a cryogen for catheter ablation of the CTI in 30 patients. This proof‐of‐concept study demonstrates that the Adagio cryoablation system is able to safely perform cryoablation of the CTI with encouraging acute and long‐term success rates, while showing favorable procedure characteristics and minimal safety concerns.

### 4.1 Acute success and procedural outcomes

We demonstrated acute efficacy in 30 out of 30 (100%) of patients, without the use of electro‐anatomic navigation, while procedure and fluoroscopy times were reduced compared with those reported in literature.^14^, ^17^, ^18^ A median of 3 + 1 bonus freeze was needed to achieve BCB and 40% of the patients showed BCB after the first freeze. In previous studies, acute success, defined as bidirectional conduction block, is reported in 85‐97% of procedures and in agreement with our findings.^2^, ^18^, ^19^, ^20^, ^21^ In general, reported procedure times for cryoablation tend to be longer than procedures using radiofrequency ablation (120–246 min and 99–198 min, respectively).^22^ Available cryoablation systems use a focal “point by point” energy delivery, requiring several freeze‐thaw cycles of many minutes.^13^, ^14^ Ablation using RF requires shorter applications and has no additional thaw time due to the lack of catheter adhesion. Using RF ablation, lines are created using a “drag and burn” method across the CTI followed by selective touch‐up to complete conduction block. The reduced procedure time (85 ± 16 min) found in our study as compared to both cryoablation and RF ablation may be attributed to the single‐shot design, using a large 37–54 mm freezing element, as well as the increased cooling power and cooling speed of near‐critical nitrogen, reducing the number and duration of freeze‐thaw cycles needed to create consistent ablation lines. For both cryoablation and RF ablation, a larger ablation tip is associated with reduced time to achieve conduction block and shorter overall procedure times.^14^, ^20^ Consequently, the small number of freezes needed to acquire conduction block further decreased the use of fluoroscopy.

### 4.2 Long‐term performance and durable conduction block

In our study, 12‐month recurrence of typical AFL on ECG was reported in 3 out of 17 AFL patients (17.6%), and in two patients conduction was confirmed during repeat EPS. A meta‐analysis performed by Pérez et al., including 9942 patients from randomized and observational studies, found a corrected AFL recurrence rate of 10.9%. Although long‐term success rate is slightly lower compared to large studies on well‐established techniques, these initial efficacy results are encouraging and demonstrate the feasibility of near‐critical nitrogen as an energy source for endocardial ablation. The first‐in‐human setting, a learning curve for the ULTC system and catheter use, and the small sample size may have hampered creating optimal long‐term durability. Although acute success was high in our cohort, the small number of freezes may have affected durable lesion formation, as the thawing phase is a pivotal part of cellular injury and late physiological reversibility may have occurred. In the 10 patients who underwent a repeat EPS in our study, we found three cases of recurrent conduction (30.0%): two symptomatic AFL patients part of the first pilot phase CC1 study, and one AF patient with no history of AFL. A study by Kuniss et al. used a 3‐month repeat EPS as confirmation of BCB in a comparison trial between an RF catheter and an early generation of cryoablation catheter. Of the 191 enrolled patients, 124 (64%) agreed to a repeat procedure. Asymptomatic conduction recurrence in the CTI was seen in 26.3% of patients in the cryoablation group and 15% in the RF group.^21^ Future, larger trials and ongoing development should demonstrate the efficacy of ultralow temperature near‐critical nitrogen as an energy source to complete durable CTI bidirectional block.

### 4.3 Safety

RF ablation of CTI‐dependent AFL is considered a first line therapy due to favorable long‐term flutter free survival and safety profile of the procedure. Serious adverse events are reported in 1‐3%.^2^, ^23^ In 2016, Patel et al. conducted a search of AFL ablations completed in the US from 2000 to 2011. Using ICD‐9 codes, they reviewed 89,638 procedures. They reported an overall mortality rate of 0.17% and an overall procedure complication rate of 3.17%. Complications included cardiac events (1.44%) as the most frequent, followed by respiratory (0.88%), vascular (0.78%), and neurologic (0.05%) complications.^22^ In the 30 subjects treated in our study, one serious adverse event was independently adjudicated as procedure related, that would be categorized in the 1.44% cardiac complication rate noted above and may not be unexpected in an AFL ablation risk analysis. With cryoablation, the effect of freezing in a very stable position may lead to vasospasm more frequently than with RF ablation. Although spasms may occur more often using cryoablation, a lower incidence of permanent damage (e.g., stenosis) is seen compared to RF ablation.^24^, ^25^ There were no unexpected procedural safety events reported. While the sample size was small, the absence of serious adverse events requiring intervention may be attributed to the low number of applications and freeze time to achieve the desired endpoint.

### 4.4 Future perspective for near critical nitrogen ablation

The development of near‐critical cooling of nitrogen paves the way for new possibilities to deliver powerful cryotherapy, down to ‐196 degrees Celsius, along a defined ablation length of small diameter devices, while using lower pressure compared to most N_2_O gas catheters and consuming much less cryogen. This technology is also very scalable and therefore there are little limitations on catheter designs, freezing lengths and position of diagnostic electrodes along the catheter.^12^ This introduces new possibilities to deliver powerful cryotherapy, using a highly customizable catheter design for multiple ablation targets.

The present study shows the ability to perform successful ablation of the CTI, but ongoing studies are directed to substantiate these findings and evaluate the capabilities to create linear and focal transmural lesions of the ablation system in other arrhythmias. Currently, the iCLAS trial (NCT04061603) is a global, single‐arm, clinical study designed to collect acute and long‐term safety and efficacy data of the 4th generation Adagio AF Cryoablation System in a larger population. 200 patients with persistent AF will be enrolled and will receive PVI and linear lesions (posterior wall and CTI) with the improved version of the ULTC system.

### 4.5 Study limitations

The main limitation of this first‐in‐human study is the exploratory nature, with a single‐arm, uncontrolled design with a small sample size. Furthermore, the study population consisted of patients suffering from AFL (CC1) and/or AF (CC2). Recurrence of AFL is therefore not a valid endpoint for CC2 patients. Acute success (stable BCB) was seen in all patients, but the protocol did not include systematic repeat EPS after 2–3 months to reassess durable BCB. Albeit multiple study visits were scheduled to investigate possible safety outcomes and symptoms, only limited scheduled repeat monitoring was performed with ECG or Holter, and additional monitoring was only performed in symptomatic patients. This may have overestimated the overall success rate. No serious adverse events leaving sequelae were seen, but larger studies are needed. Finally, results may be influenced by the learning curve of both centers and ongoing trials are needed to confirm safety and long‐term efficacy.

## 5 CONCLUSION

In this first‐in‐human, thirty‐patient clinical study, the safety and performance results demonstrate the capabilities of ultralow temperature near‐critical nitrogen as an energy source for ablation of the CTI. One transient procedure‐related adverse event (coronary artery spasm) was reported. Acute success was achieved in all subjects, while 82.4% remained free of AFL recurrence at 12‐month follow‐up. The ultralow temperatures achieved, the lower number and shorter duration of freezes compared to other cryoablation technologies suggest a high potential for this novel energy source. Future studies will evaluate the capabilities of the system in larger sample sizes and other arrhythmias.

## CONFLICT OF INTERESTS

Tom J. R. De Potter and Lucas V. A. Boersma report consultancy fees for Adagio. Alexander Babkin and David Cabrita acknowledge salary and stock options compensation as Adagio Medical employees. Alexander Babkin and David Cabrita acknowledge to be authors of Adagio Medical patents related to catheters using the technology described. David Cabrita acknowledges to be a current employee at Medtronic, a company that may closely compete with the devices used in this study.
